# Pseudoprogression Associated With T-cell Infiltration During Tarlatamab Therapy for Small-Cell Lung Cancer: A Case Report

**DOI:** 10.7759/cureus.103393

**Published:** 2026-02-10

**Authors:** Tomoyuki Ikeuchi, Mitsuhiro Yamamoto, Hirokazu Toge, Naoto Burioka, Akira Yamasaki

**Affiliations:** 1 Department of Respiratory Medicine, National Hospital Organization (NHO) Yonago Medical Center, Yonago, JPN; 2 Faculty of Medicine, Tottori University, Yonago, JPN; 3 Division of Respiratory Medicine and Rheumatology, Department of Multidisciplinary Internal Medicine, School of Medicine, Faculty of Medicine, Tottori University, Yonago, JPN

**Keywords:** bispecific t-cell engager, immunotherapy, pseudoprogression, small-cell lung cancer, tarlatamab, t-cell infiltration

## Abstract

Tarlatamab is a bispecific T-cell engager immunotherapy that targets delta-like ligand 3 and CD3. We report a case of pseudoprogression (PsPD) in a patient with small-cell lung cancer treated with tarlatamab. Due to its mechanism, tarlatamab is known to induce T-cell infiltration into tumors. In PsPD, the tumor transiently enlarges because of massive T-cell accumulation within the tumor. In this case, the peripheral blood lymphocyte count decreased, and the interleukin-6 level was markedly elevated, which was thought to reflect T-cell accumulation in the tumor. These trends in the blood tests may be useful in easily differentiating PsPD from true progressive disease during tarlatamab treatment.

## Introduction

Transient radiologic tumor progression from baseline may be observed in patients with lung cancer receiving immune checkpoint inhibitor treatment. This phenomenon is known as pseudoprogression (PsPD) [1]. The incidence of PsPD in non-small-cell lung cancer has been reported to be 0-5% [2]. PsPD has also been reported in patients with small-cell lung cancer (SCLC) since immune checkpoint inhibitors became available for SCLC [3]. Although the detailed mechanism of PsPD during immune checkpoint inhibitor therapy remains unclear, lymphocyte infiltration into tumors has been suggested as a possible contributing factor [1]. Distinguishing PsPD from true progressive disease (TPD) during immunotherapy is critical because it affects subsequent treatment strategies. However, to our knowledge, there are no unified diagnostic criteria for differentiating the two, which remains an ongoing challenge [4].

Tarlatamab was developed to treat SCLC through a novel immune mechanism [5]. Tarlatamab is a bispecific T-cell engager (BiTE) immunotherapy that targets delta-like ligand 3 and CD3. Microscopic T-cell infiltration into tumors has been observed in preclinical animal studies, as it promotes T-cell activation and directs T cells toward tumor cells [6]. BiTE agents were first used to treat hematological malignancies. Some cases of PsPD during BiTE therapy for hematologic malignancies have been reported under the term “tumor flare reaction” [7,8]. In cases of diffuse large B-cell lymphoma, a histopathological evaluation was performed to detect worsening skin lesions [8]. Similar to preclinical animal studies, CD4+ and CD8+ lymphocytes were clustered around the tumor.

Here, we report a case of PsPD during tarlatamab treatment, which has been approved for SCLC treatment. This report describes the clinical course and characteristics of tarlatamab-induced PsPD.

## Case presentation

A 73-year-old man presented with right back pain and anorexia in June 2024. He had a smoking history of 10 cigarettes per day for 40 years. He was diagnosed with extensive-stage SCLC (cT3N2M1c (multiple brain metastases), stage IVB). The patient received cisplatin, etoposide, and durvalumab as first-line therapy, followed by maintenance therapy with durvalumab. Due to the progression of multiple brain metastases, the patient was treated with amrubicin as second-line therapy. Third-line topotecan therapy was administered, but progressive disease was determined owing to worsening pulmonary lesions.

**Figure 1:**
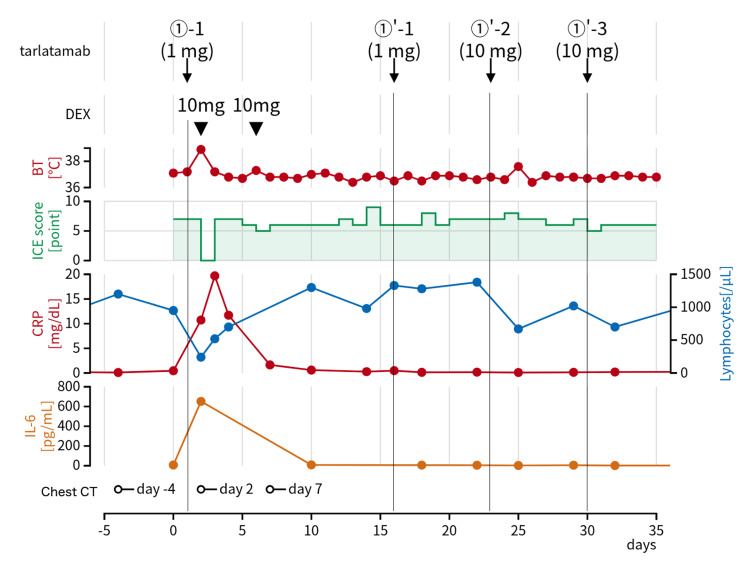
Clinical course following tarlatamab administration. On day two, the patient developed a fever of 38.9°C with a decline in the ICE score. These findings were consistent with those of cytokine release syndrome and ICANS, and dexamethasone equivalent to 10 mg was administered. On day six, a low-grade fever of 37.3°C occurred with the ICE score decreasing to 5 points, prompting the administration of dexamethasone equivalent to 10 mg for suspected ICANS. Laboratory data showed that peripheral blood lymphocyte count was decreased from 950/μL on day zero to 240/μL on day two, and interleukin-6 was markedly elevated from 7.6 pg/mL on day zero to 652 pg/mL on day two. C-reactive protein reached a peak value of 19.6 mg/dL on day three, with a one-day delay. BT: body temperature; CRP: C-reactive protein; DEX: dexamethasone; ICANS: immune effector cell-associated neurotoxicity syndrome; ICE: immune effector cell-associated encephalopathy; IL-6: interleukin-6

**Figure 2:**
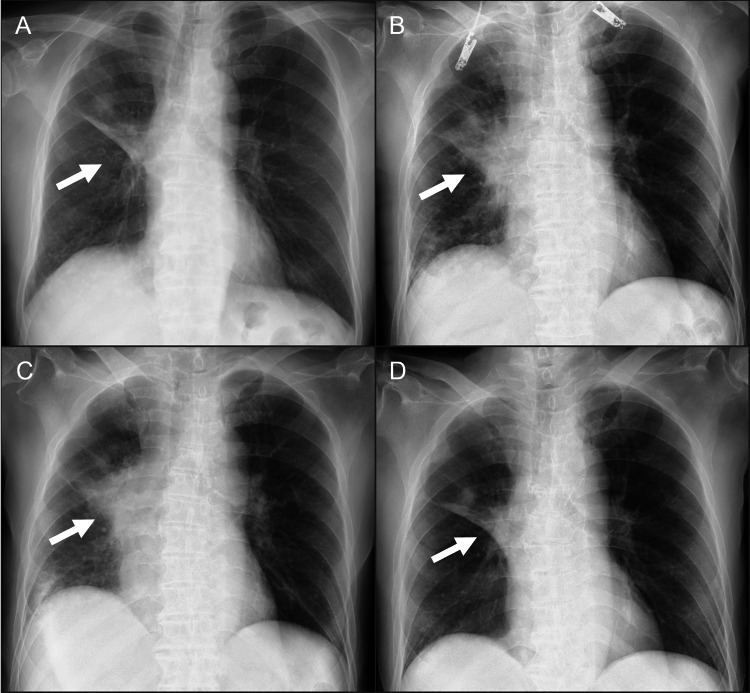
Radiographic course on chest X-ray. The right hilar mass increased in size on days two (B) and four (C). On day seven (D), the mass had decreased in size. Arrows indicate the areas of interest. (A) Chest radiograph on day zero. (B) Chest radiograph on day two. (C) Chest radiograph on day four. (D) Chest radiograph on day seven.

**Figure 3:**
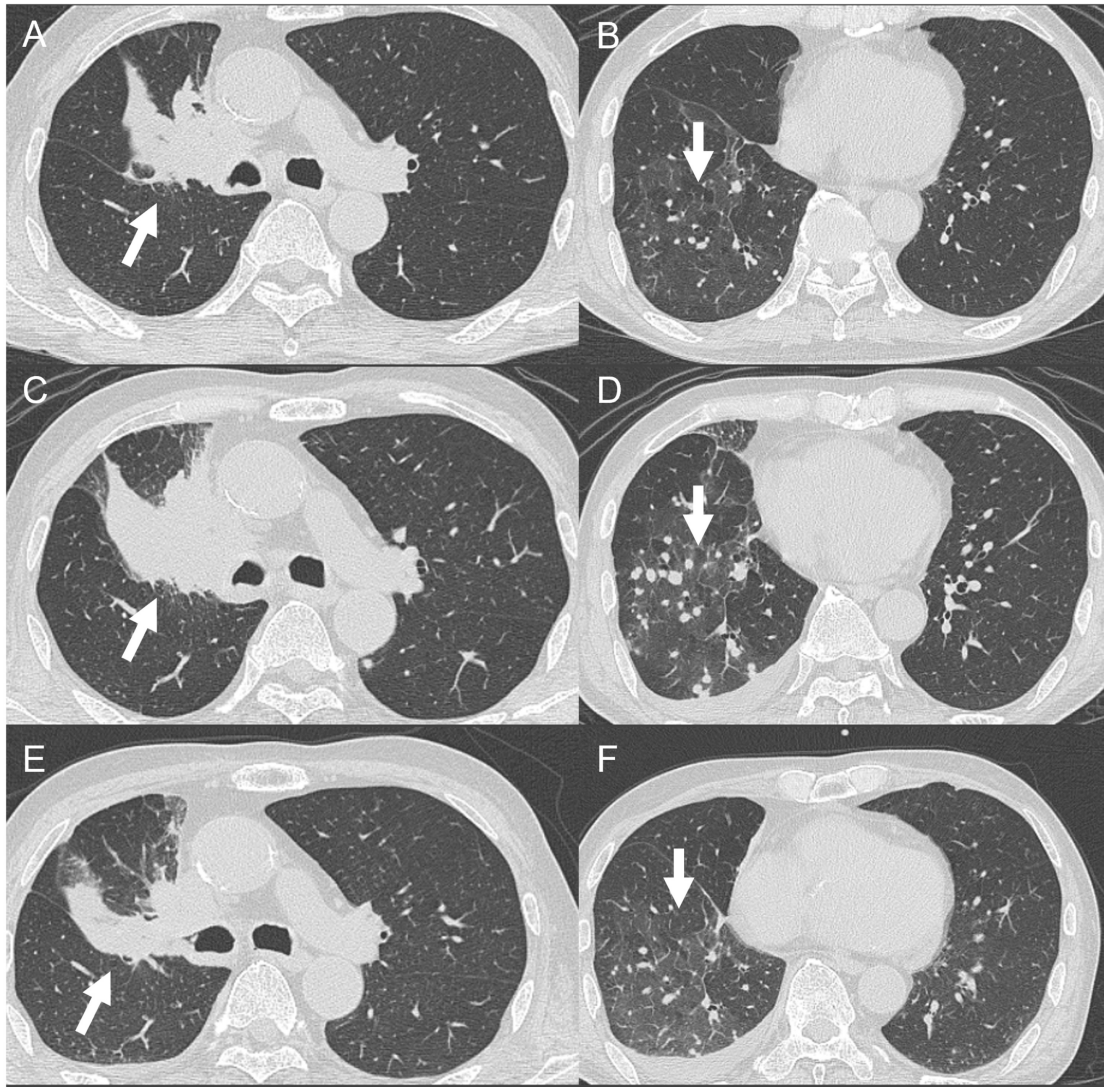
Radiographic course on chest CT scan. Right hilar mass and right lower lobe metastases enlarged transiently on day two (C, D) and subsequently decreased in size on day seven (E, F). Arrows indicate the areas of interest. (A) CT image at the carinal level on day -four. (B) CT image of the lower lobe level on day -four. (C) CT image at the carinal level on day two. (D) CT image at the lower lobe level on day two. (E) CT image at the carinal level on day seven. (F) CT image at the lower lobe level on day seven.

Fourth-line therapy with tarlatamab was initiated in October 2025. Tarlatamab 1 mg was administered on day one per the protocol. Before tarlatamab administration, the patient had an immune effector cell-associated encephalopathy (ICE) score of 7 points, with incorrect responses to questions regarding the current date and calculations. On day two, he developed a fever of 38.9°C (Figure 1). On the same day, the patient developed difficulty speaking and moving his limbs. The ICE score decreased to 0 points. He was graded as having cytokine release syndrome (CRS) Grade 2 and immune effector cell-associated neurotoxicity syndrome (ICANS) Grade 3. Intravenous dexamethasone equivalent to 10 mg was administered. Laboratory tests showed an elevation in C-reactive protein (CRP) to 10.7 mg/dL, and a decrease in the peripheral blood lymphocyte count to 240/μL. The interleukin-6 (IL-6) level was markedly elevated to 652 pg/mL. Chest radiography showed marked enlargement of the right hilar mass compared to that before tarlatamab administration (Figures 2A, 2B). Chest CT also demonstrated enlargement of the right hilar mass and multiple metastatic pulmonary nodules in the right lower lobe compared to baseline (Figures 3A-3D). After dexamethasone administration, the body temperature decreased to 37.2°C, and the ICE score recovered to 7 points. On day four, the hilar mass remained enlarged on chest radiography (Figure 2C). On day six, the ICE score decreased again to 5 points, accompanied by a low-grade fever (37.2℃). This was determined to be ICANS Grade 2, and intravenous dexamethasone equivalent to 10 mg was administered. On day seven, chest radiography revealed a reduction of the hilar mass (Figure 2D). Chest CT confirmed reduction of both the hilar mass and right lower lobe pulmonary metastases (Figures 3E, 3F). These radiographic findings of improvement led to the diagnosis of PsPD. On day 10, the IL-6 level improved to 8.1 pg/mL and returned to pre-tarlatamab levels. CRP and peripheral blood lymphocyte count also returned to pre-tarlatamab levels. Because the ICE score decreased to 0 after the administration of tarlatamab 1 mg, tarlatamab 10 mg was not administered on day eight. Tarlatamab 1 mg was restarted on day 15 after the patient’s condition stabilized. Subsequently, tarlatamab was administered as per protocol without CRS or ICANS.

## Discussion

Based on the clinical course, this case was diagnosed as PsPD. PubMed was searched for previously reported cases of tarlatamab-induced PsPD. The three identified cases are listed in Table 1 [9-11]. Including our case, radiographic progression was observed in all cases within the very early period of one week after initial administration. Radiographic improvement was also observed relatively early (within two weeks of administration). The administration of BiTE agents has been shown to induce T-cell infiltration around tumors in both preclinical animal studies [6] and humans [8]. Therefore, we hypothesized that transient radiographic tumor enlargement in tarlatamab-induced PsPD results from T-cell infiltration into the tumors. It is presumed that subsequent tumor reduction occurs as BiTE-engaged T cells attack the tumor cells.

**Table 1:** Reported cases of PsPD after tarlatamab administration. CRS: cytokine release syndrome; ICANS: immune effector cell-associated neurotoxicity syndrome; PsPD: pseudoprogression

Study	Sex	Age	Treatment line	Time of PsPD onset	Time of improvement	Progressive lesions	Adverse events	Lymphocyte count at PsPD onset
Shijubou et al., 2025 [9]	Female	60s	Fourth-line	Day 2	Day 16	Enlargement of brain metastases, peritumoral edema	Negative	Not listed
Nakano et al., 2025 Aug. [10]	Female	59	Third-line	Day 7	Day 12	Enlargement of the primary tumor, new pleural effusion	CRS (G2)	Decrease
Kushima et al., 2025 Nov. [11]	Male	77	Sixth-line	Day 3	Day 14	Enlargement of the primary tumor, diffuse infiltrates in the lung	CRS (G2)	Not listed
Present case	Male	73	Fourth-line	Day 2	Day 7	Enlargement of the primary tumor, enlargement of the lung metastases	CRS (G2), ICANS (G3)	Decrease

In this case, the peripheral blood lymphocyte count decreased at the onset of PsPD. We speculate that this apparent decrease in the peripheral blood lymphocyte count resulted from the massive recruitment of lymphocytes from the peripheral blood to the tumor microenvironment over a short period. A similar decrease in the peripheral blood lymphocyte count during PsPD has been reported previously [10]. Blood IL-6 levels were also markedly elevated at the onset of PsPD, likely due to inflammatory cytokine secretion resulting from the massive mobilization of T cells to tumor cells.

When radiographic progression is observed after tarlatamab administration, distinguishing PsPD from TPD is challenging. Furthermore, cases of rapid disease progression following tarlatamab administration have been reported [12], with regulatory T-cell amplification suggested as a potential mechanism. Therefore, early differentiation between PsPD and TPD is critical for determining subsequent treatment strategies. In this case, decreased peripheral blood lymphocyte count and elevated IL-6 levels were observed during the early phase of PsPD. These findings likely reflect massive T-cell mobilization to the tumor microenvironment and may serve as useful biomarkers for differentiating PsPD from TPD.

## Conclusions

Tarlatamab administration may cause PsPD, which is characterized by transient tumor enlargement. Tarlatamab-induced PsPD occurred within one week after administration. Tumor reduction also occurred relatively early, within two to three weeks of administration. In this case, the peripheral blood lymphocyte count decreased, and blood IL-6 levels were elevated at the onset of PsPD. These responses likely reflect the result of massive T-cell infiltration into the tumor. When radiographic progression is observed following tarlatamab administration, decreased peripheral lymphocyte count and markedly elevated IL-6 may be useful in differentiating PsPD from TPD. However, this is an observation from a single case, and further investigation is needed to determine whether similar trends will be observed in future cases.​​​​​​
